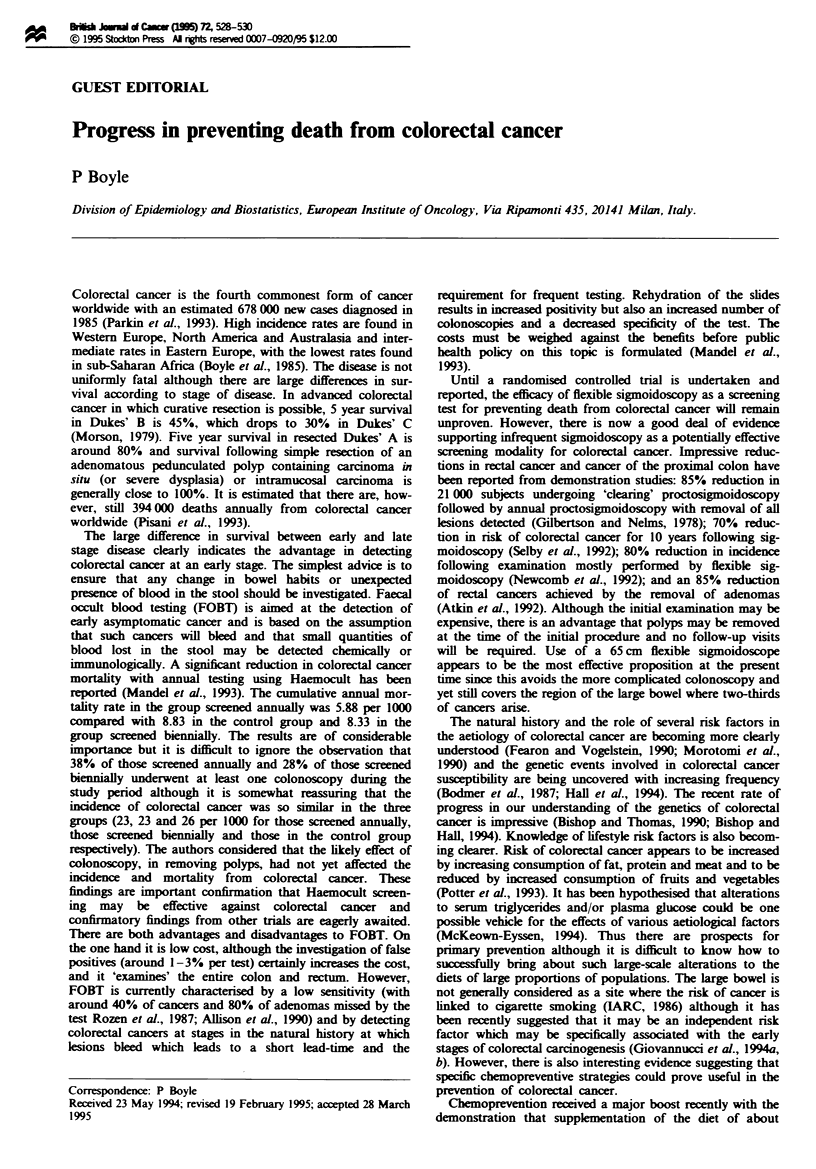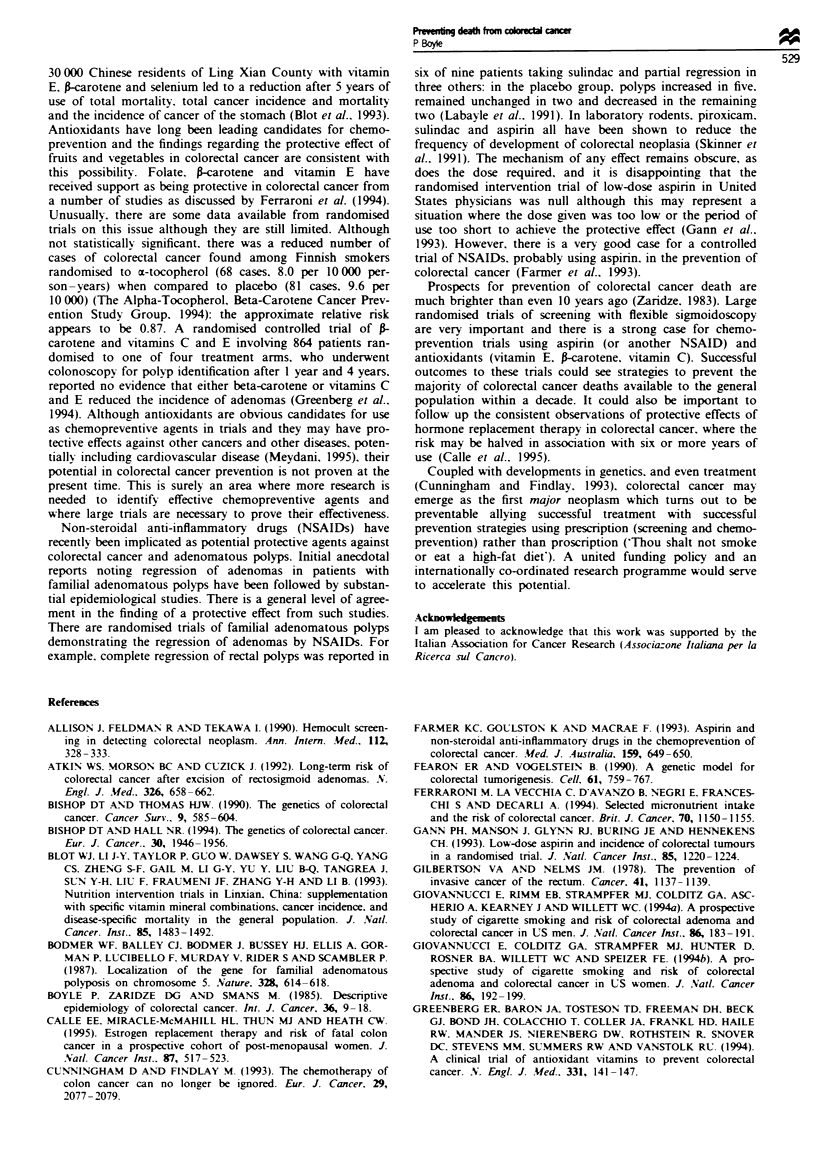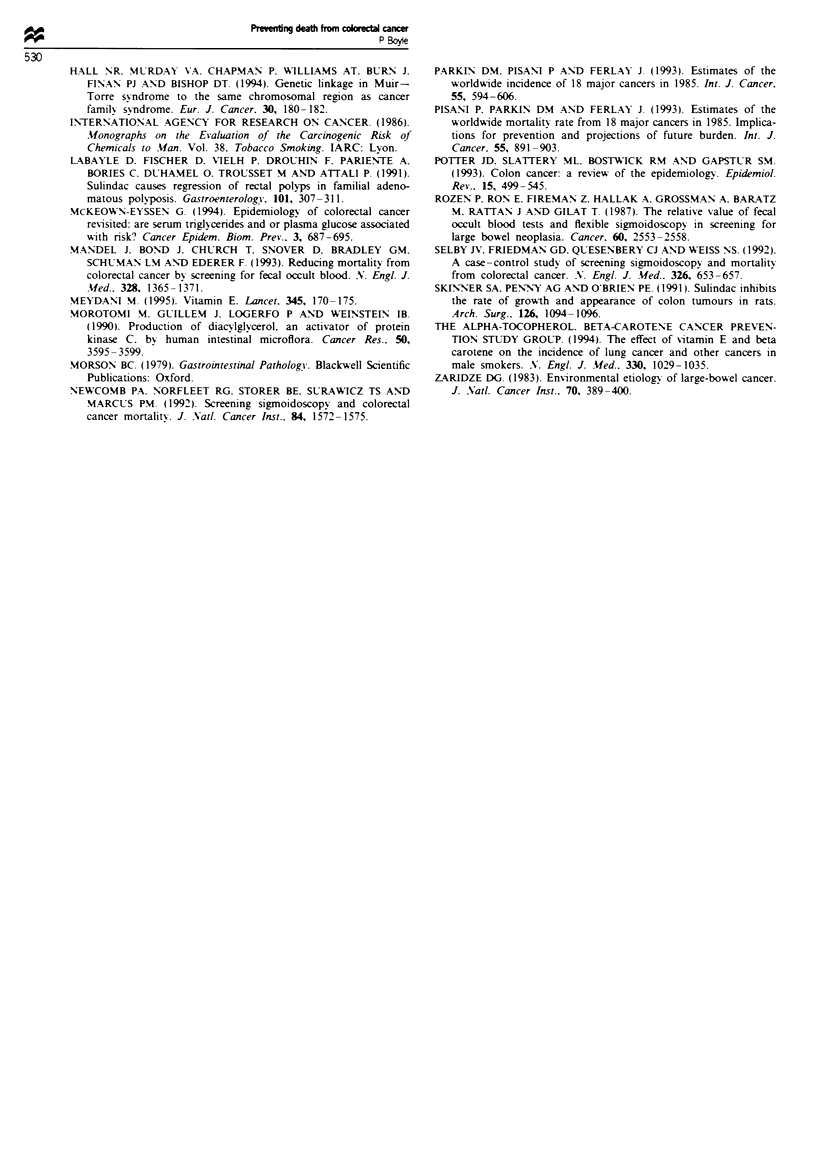# Progress in preventing death from colorectal cancer.

**DOI:** 10.1038/bjc.1995.368

**Published:** 1995-09

**Authors:** P. Boyle


© 1995 Stockton Press All rights reserved 0007-0920/95 $12.00

GUEST EDITORIAL

### Progress in preventing death from colorectal cancer

P Boyle

*Division of Epidemiology and Biostatistics, European Institute of Oncology, Via Ripamonti 435, 20141 Milan, Italy.*

Colorectal cancer is the fourth commonest form of cancer worldwide with an estimated 678 000 new cases diagnosed in 1985 (Parkin *et al.*, 1993). High incidence rates are found in Western Europe, North America and Australasia and intermediate rates in Eastern Europe, with the lowest rates found in sub-Saharan Africa (Boyle *et al.*, 1985). The disease is not uniformly fatal although there are large differences in survival according to stage of disease. In advanced colorectal cancer in which curative resection is possible, 5 year survival in Dukes' B is 45%, which drops to 30% in Dukes' C (Morson, 1979). Five year survival in resected Dukes' A is around 80% and survival following simple resection of an adenomatous pedunculated polyp containing carcinoma *in situ* (or severe dysplasia) or intramucosal carcinoma is generally close to 100%. It is estimated that there are, however, still 394 000 deaths annually from colorectal cancer worldwide (Pisani *et al.*, 1993).

The large difference in survival between early and late stage disease clearly indicates the advantage in detecting colorectal cancer at an early stage. The simplest advice is to ensure that any change in bowel habits or unexpected presence of blood in the stool should be investigated. Faecal occult blood testing (FOBT) is aimed at the detection of early asymptomatic cancer and is based on the assumption that such cancers will bleed and that small quantities of blood lost in the stool may be detected chemically or immunologically. A significant reduction in colorectal cancer mortality with annual testing using Haemocult has been reported (Mandel *et al.*, 1993). The cumulative annual mortality rate in the group screened annually was 5.88 per 1000 compared with 8.83 in the control group and 8.33 in the group screened biennially. The results are of considerable importance but it is difficult to ignore the observation that 38% of those screened annually and 28% of those screened biennially underwent at least one colonoscopy during the study period although it is somewhat reassuring that the incidence of colorectal cancer was so similar in the three groups (23, 23 and 26 per 1000 for those screened annually, those screened biennially and those in the control group respectively). The authors considered that the likely effect of colonoscopy, in removing polyps, had not yet affected the incidence and mortality from colorectal cancer. These findings are important confirmation that Haemocult screening may be effective against colorectal cancer and confirmatory findings from other trials are eagerly awaited. There are both advantages and disadvantages to FOBT. On the one hand it is low cost, although the investigation of false positives (around 1-3% per test) certainly increases the cost, and it 'examines' the entire colon and rectum. However, FOBT is currently characterised by a low sensitivity (with around 40% of cancers and 80% of adenomas missed by the test Rozen *et al.*, 1987; Allison *et al.*, 1990) and by detecting colorectal cancers at stages in the natural history at which lesions bleed which leads to a short lead-time and the requirement for frequent testing. Rehydration of the slides results in increased positivity but also an increased number of colonoscopies and a decreased specificity of the test. The costs must be weighed against the benefits before public health policy on this topic is formulated (Mandel *et al.*, 1993).

Until a randomised controlled trial is undertaken and reported, the efficacy of flexible sigmoidoscopy as a screening test for preventing death from colorectal cancer will remain unproven. However, there is now a good deal of evidence supporting infrequent sigmoidoscopy as a potentially effective screening modality for colorectal cancer. Impressive reductions in rectal cancer and cancer of the proximal colon have been reported from demonstration studies: 85% reduction in 21 000 subjects undergoing 'clearing' proctosigmoidoscopy followed by annual proctosigmoidoscopy with removal of all lesions detected (Gilbertson and Nelms, 1978); 70% reduction in risk of colorectal cancer for 10 years following sigmoidoscopy (Selby *et al.*, 1992); 80% reduction in incidence following examination mostly performed by flexible sigmoidoscopy (Newcomb *et al.*, 1992); and an 85% reduction of rectal cancers achieved by the removal of adenomas (Atkin *et al.*, 1992). Although the initial examination may be expensive, there is an advantage that polyps may be removed at the time of the initial procedure and no follow-up visits will be required. Use of a 65 cm flexible sigmoidoscope appears to be the most effective proposition at the present time since this avoids the more complicated colonoscopy and yet still covers the region of the large bowel where two-thirds of cancers arise.

The natural history and the role of several risk factors in the aetiology of colorectal cancer are becoming more clearly understood (Fearon and Vogelstein, 1990; Morotomi *et al.*, 1990) and the genetic events involved in colorectal cancer susceptibility are being uncovered with increasing frequency (Bodmer *et al.*, 1987; Hall *et al.*, 1994). The recent rate of progress in our understanding of the genetics of colorectal cancer is impressive (Bishop and Thomas, 1990; Bishop and Hall, 1994). Knowledge of lifestyle risk factors is also becoming clearer. Risk of colorectal cancer appears to be increased by increasing consumption of fat, protein and meat and to be reduced by increased consumption of fruits and vegetables (Potter *et al.*, 1993). It has been hypothesised that alterations to serum triglycerides and/or plasma glucose could be one possible vehicle for the effects of various aetiological factors (McKeown-Eyssen, 1994). Thus there are prospects for primary prevention although it is difficult to know how to successfully bring about such large-scale alterations to the diets of large proportions of populations. The large bowel is not generally considered as a site where the risk of cancer is linked to cigarette smoking (IARC, 1986) although it has been recently suggested that it may be an independent risk factor which may be specifically associated with the early stages of colorectal carcinogenesis (Giovannucci *et al.*, 1994*a*, *b*). However, there is also interesting evidence suggesting that specific chemopreventive strategies could prove useful in the prevention of colorectal cancer.

Chemoprevention received a major boost recently with the demonstration that supplementation of the diet of about

Correspondence: P Boyle
Received 23 May 1994; revised 19 February 1995; accepted 28 March 1995

30 000 Chinese residents of Ling Xian County with vitamin E, β-carotene and selenium led to a reduction after 5 years of use of total mortality, total cancer incidence and mortality and the incidence of cancer of the stomach (Blot *et al.*, 1993). Antioxidants have long been leading candidates for chemoprevention and the findings regarding the protective effect of fruits and vegetables in colorectal cancer are consistent with this possibility. Folate, β-carotene and vitamin E have received support as being protective in colorectal cancer from a number of studies as discussed by Ferraroni *et al.* (1994). Unusually, there are some data available from randomised trials on this issue although they are still limited. Although not statistically significant, there was a reduced number of cases of colorectal cancer found among Finnish smokers randomised to α-tocopherol (68 cases, 8.0 per 10 000 person-years) when compared to placebo (81 cases, 9.6 per 10 000) (The Alpha-Tocopherol, Beta-Carotene Cancer Prevention Study Group, 1994): the approximate relative risk appears to be 0.87. A randomised controlled trial of β-carotene and vitamins C and E involving 864 patients randomised to one of four treatment arms, who underwent colonoscopy for polyp identification after 1 year and 4 years, reported no evidence that either beta-carotene or vitamins C and E reduced the incidence of adenomas (Greenberg *et al.*, 1994). Although antioxidants are obvious candidates for use as chemopreventive agents in trials and they may have protective effects against other cancers and other diseases, potentially including cardiovascular disease (Meydani, 1995), their potential in colorectal cancer prevention is not proven at the present time. This is surely an area where more research is needed to identify effective chemopreventive agents and where large trials are necessary to prove their effectiveness.

Non-steroidal anti-inflammatory drugs (NSAIDs) have recently been implicated as potential protective agents against colorectal cancer and adenomatous polyps. Initial anecdotal reports noting regression of adenomas in patients with familial adenomatous polyps have been followed by substantial epidemiological studies. There is a general level of agreement in the finding of a protective effect from such studies. There are randomised trials of familial adenomatous polyps demonstrating the regression of adenomas by NSAIDs. For example, complete regression of rectal polyps was reported in six of nine patients taking sulindac and partial regression in three others: in the placebo group, polyps increased in five, remained unchanged in two and decreased in the remaining two (Labayle *et al.*, 1991). In laboratory rodents, piroxicam, sulindac and aspirin all have been shown to reduce the frequency of development of colorectal neoplasia (Skinner *et al.*, 1991). The mechanism of any effect remains obscure, as does the dose required, and it is disappointing that the randomised intervention trial of low-dose aspirin in United States physicians was null although this may represent a situation where the dose given was too low or the period of use too short to achieve the protective effect (Gann *et al.*, 1993). However, there is a very good case for a controlled trial of NSAIDs, probably using aspirin, in the prevention of colorectal cancer (Farmer *et al.*, 1993).

Prospects for prevention of colorectal cancer death are much brighter than even 10 years ago (Zaridze, 1983). Large randomised trials of screening with flexible sigmoidoscopy are very important and there is a strong case for chemoprevention trials using aspirin (or another NSAID) and antioxidants (vitamin E, β-carotene, vitamin C). Successful outcomes to these trials could see strategies to prevent the majority of colorectal cancer deaths available to the general population within a decade. It could also be important to follow up the consistent observations of protective effects of hormone replacement therapy in colorectal cancer, where the risk may be halved in association with six or more years of use (Calle *et al.*, 1995).

Coupled with developments in genetics, and even treatment (Cunningham and Findlay, 1993), colorectal cancer may emerge as the first *major* neoplasm which turns out to be preventable allying successful treatment with successful prevention strategies using prescription (screening and chemoprevention) rather than proscription ('Thou shalt not smoke or eat a high-fat diet'). A united funding policy and an internationally co-ordinated research programme would serve to accelerate this potential.

**Acknowledgements**

I am pleased to acknowledge that this work was supported by the Italian Association for Cancer Research (*Associazone Italiana per la Ricerca sul Cancro*).

**References**

ALLISON J, FELDMAN R AND TEKAWA I. (1990). Hemocult screening in detecting colorectal neoplasm. *Ann. Intern. Med.*, **112**, 328-333.

ATKIN WS, MORSON BC AND CUZICK J. (1992). Long-term risk of colorectal cancer after excision of rectosigmoid adenomas. *N. Engl. J. Med.*, **326**, 658-662.

BISHOP DT AND THOMAS HJW. (1990). The genetics of colorectal cancer. *Cancer Surv.*, **9**, 585-604.

BISHOP DT AND HALL NR. (1994). The genetics of colorectal cancer. *Eur. J. Cancer.*, **30**, 1946-1956.

BLOT WJ, LI J-Y, TAYLOR P, GUO W, DAWSEY S, WANG G-Q, YANG CS, ZHENG S-F, GAIL M, LI G-Y, YU Y, LIU B-Q, TANGREA J, SUN Y-H, LIU F, FRAUMENI JF, ZHANG Y-H AND LI B. (1993). Nutrition intervention trials in Linxian, China: supplementation with specific vitamin mineral combinations, cancer incidence, and disease-specific mortality in the general population. *J. Natl. Cancer. Inst.*, **85**, 1483-1492.

BODMER WF, BALLEY CJ, BODMER J, BUSSEY HJ, ELLIS A, GORMAN P, LUCIBELLO F, MURDAY V, RIDER S AND SCAMBLER P. (1987). Localization of the gene for familial adenomatous polyposis on chromosome 5. *Nature*, **328**, 614-618.

BOYLE P, ZARIDZE DG AND SMANS M. (1985). Descriptive epidemiology of colorectal cancer. *Int. J. Cancer*, **36**, 9-18.

CALLE EE, MIRACLE-McMAHILL HL, THUN MJ AND HEATH CW. (1995). Estrogen replacement therapy and risk of fatal colon cancer in a prospective cohort of post-menopausal women. *J. Natl. Cancer Inst.*, **87**, 517-523.

CUNNINGHAM D AND FINDLAY M. (1993). The chemotherapy of colon cancer can no longer be ignored. *Eur. J. Cancer*, **29**, 2077-2079.

FARMER KC, GOULSTON K AND MACRAE F. (1993). Aspirin and non-steroidal anti-inflammatory drugs in the chemoprevention of colorectal cancer. *Med. J. Australia*, **159**, 649-650.

FEARON ER AND VOGELSTEIN B. (1990). A genetic model for colorectal tumorigenesis. *Cell*, **61**, 759-767.

FERRARONI M, LA VECCHIA C, D'AVANZO B, NEGRI E, FRANCESCHI S AND DECARLI A. (1994). Selected micronutrient intake and the risk of colorectal cancer. *Brit. J. Cancer*, **70**, 1150-1155.

GANN PH, MANSON J, GLYNN RJ, BURING JE AND HENNEKENS CH. (1993). Low-dose aspirin and incidence of colorectal tumours in a randomised trial. *J. Natl. Cancer Inst.*, **85**, 1220-1224.

GILBERTSON VA AND NELMS JM. (1978). The prevention of invasive cancer of the rectum. *Cancer*, **41**, 1137-1139.

GIOVANNUCCI E, RIMM EB, STRAMPFER MJ, COLDITZ GA, ASCHERIO A, KEARNEY J AND WILLETT WC. (1994*a*). A prospective study of cigarette smoking and risk of colorectal adenoma and colorectal cancer in US men. *J. Natl. Cancer Inst.*, **86**, 183-191.

GIOVANNUCCI E, COLDITZ GA, STRAMPFER MJ, HUNTER D, ROSNER BA, WILLETT WC AND SPEIZER FE. (1994*b*). A prospective study of cigarette smoking and risk of colorectal adenoma and colorectal cancer in US women. *J. Natl. Cancer Inst.*, **86**, 192-199.

GREENBERG ER, BARON JA, TOSTESON TD, FREEMAN DH, BECK GJ, BOND JH, COLACCHIO T, COLLER JA, FRANKL HD, HAILE RW, MANDER JS, NIERENBERG DW, ROTHSTEIN R, SNOVER DC, STEVENS MM, SUMMERS RW AND VANSTOLK RU. (1994). A clinical trial of antioxidant vitamins to prevent colorectal cancer. *N. Engl. J. Med.*, **331**, 141-147.

HALL NR, MURDAY VA, CHAPMAN P, WILLIAMS AT, BURN J, FINAN PJ AND BISHOP DT. (1994). Genetic linkage in Muir–Torre syndrome to the same chromosomal region as cancer family syndrome. *Eur. J. Cancer*, **30**, 180–182.

INTERNATIONAL AGENCY FOR RESEARCH ON CANCER. (1986). *Monographs on the Evaluation of the Carcinogenic Risk of Chemicals to Man*. Vol. 38, *Tobacco Smoking*. IARC: Lyon.

LABAYLE D, FISCHER D, VIELH P, DROUHIN F, PARIENTE A, BORIES C, DUHAMEL O, TROUSSET M AND ATTALI P. (1991). Sulindac causes regression of rectal polyps in familial adenomatous polyposis. *Gastroenterology*, **101**, 307–311.

McKEOWN-EYSSEN G. (1994). Epidemiology of colorectal cancer revisited: are serum triglycerides and or plasma glucose associated with risk? *Cancer Epidem. Biom. Prev.*, **3**, 687–695.

MANDEL J, BOND J, CHURCH T, SNOVER D, BRADLEY GM, SCHUMAN LM AND EDERER F. (1993). Reducing mortality from colorectal cancer by screening for fecal occult blood. *N. Engl. J. Med.*, **328**, 1365–1371.

MEYDANI M. (1995). Vitamin E. *Lancet*, **345**, 170–175.

MOROTOMI M, GUILLEM J, LOGERFO P AND WEINSTEIN IB. (1990). Production of diacylglycerol, an activator of protein kinase C, by human intestinal microflora. *Cancer Res.*, **50**, 3595–3599.

MORSON BC. (1979). *Gastrointestinal Pathology*. Blackwell Scientific Publications: Oxford.

NEWCOMB PA, NORFLEET RG, STORER BE, SURAWICZ TS AND MARCUS PM. (1992). Screening sigmoidoscopy and colorectal cancer mortality. *J. Natl. Cancer Inst.*, **84**, 1572–1575.

PARKIN DM, PISANI P AND FERLAY J. (1993). Estimates of the worldwide incidence of 18 major cancers in 1985. *Int. J. Cancer*, **55**, 594–606.

PISANI P, PARKIN DM AND FERLAY J. (1993). Estimates of the worldwide mortality rate from 18 major cancers in 1985. Implications for prevention and projections of future burden. *Int. J. Cancer*, **55**, 891–903.

POTTER JD, SLATTERY ML, BOSTWICK RM AND GAPSTUR SM. (1993). Colon cancer: a review of the epidemiology. *Epidemiol. Rev.*, **15**, 499–545.

ROZEN P, RON E, FIREMAN Z, HALLAK A, GROSSMAN A, BARATZ M, RATTAN J AND GILAT T. (1987). The relative value of fecal occult blood tests and flexible sigmoidoscopy in screening for large bowel neoplasia. *Cancer*, **60**, 2553–2558.

SELBY JV, FRIEDMAN GD, QUESENBERY CJ AND WEISS NS. (1992). A case–control study of screening sigmoidoscopy and mortality from colorectal cancer. *N. Engl. J. Med.*, **326**, 653–657.

SKINNER SA, PENNY AG AND O'BRIEN PE. (1991). Sulindac inhibits the rate of growth and appearance of colon tumours in rats. *Arch. Surg.*, **126**, 1094–1096.

THE ALPHA-TOCOPHEROL, BETA-CAROTENE CANCER PREVENTION STUDY GROUP. (1994). The effect of vitamin E and beta carotene on the incidence of lung cancer and other cancers in male smokers. *N. Engl. J. Med.*, **330**, 1029–1035.

ZARIDZE DG. (1983). Environmental etiology of large-bowel cancer. *J. Natl. Cancer Inst.*, **70**, 389–400.